# Immunoregulatory Effects of Subcutaneous Immunotherapy on Lymphocyte Subgroups and Cytokines in Children with Asthma

**DOI:** 10.1155/2019/7024905

**Published:** 2019-10-13

**Authors:** Yu-ting He, Ying Zhou, Qi Shao, Cong Gan, Meng Chen, Yu-ling Bao, Hai-yan Gu, Shu-lan Zhang, Yubao Cui, Man Tian

**Affiliations:** ^1^Respiratory Department, Children's Hospital of Nanjing Medical University, Nanjing, 210008 Jiangsu Province, China; ^2^Department of Pediatrics Laboratory, Wuxi People's Hospital Affiliated to Nanjing Medical University, Wuxi 214023, China; ^3^Department of Clinical Laboratory, Wuxi People's Hospital Affiliated to Nanjing Medical University, Wuxi 214023, China

## Abstract

**Objective:**

Asthma is a syndrome that incorporates many immune phenotypes. The immunologic effects of subcutaneous immunotherapy (SCIT) exerts on allergic asthma remain still largely unknown. Here, we investigated the effects of SCIT on cytokine production and peripheral blood levels of lymphocyte subtypes in children with mite-induced moderate and severe allergic asthma.

**Methods:**

The study included 60 kids with mite-induced allergic asthma from 5 to 10 years old. All subjects had received antiasthmatic pharmacologic for 3 months at baseline. Half of the children were treated with SCIT combined with pharmacologic treatment named the SCIT group and the other half only with pharmacologic therapy named the no-SCIT group. Total asthma symptom score (TASS) and total medication score (TMS) were recorded. Flow cytometry was used to identify lymphocyte subtypes: type 2 innate lymphocytes (ILC2s), type 1 (Th1) and type 2 (Th2) helper T cells, T helper 17 (Th17) cells, and regulatory T (Treg) cells. ELISA, flow cytometry, and cytometric bead array were used to assess cytokines IL-13, IFN-*γ*, IL-4, IL-17, and TGF-*β*, at baseline and 3 and 6 months after study treatment in both groups of patients.

**Results:**

Both groups can significantly improve clinical symptoms in children with asthma. SCIT can significantly reduce asthma medication after 6 months of treatment. SCIT induced a significantly higher and progressive reduction in ILC2 percentage and IL-13 levels after 3 and 6 months of treatment compared with baseline and compared with no-SCIT patients. Significant differences were detected in the Th1/Th2 cell ratio and IFN-*γ*/IL-4 cytokine ratio between groups after 6 months of treatment. Similarly, the Th17/Treg ratio and IL-17/TGF-*β* ratio in the SCIT group were much lower than those in the no-SCIT group after 3-6 months of treatment.

**Conclusion:**

SCIT is a promising option to reduce the percentage of ILC2 and regulate Th1/Th2 and Th17/Treg immune balance in the peripheral blood of children with asthma.

## 1. Background

Bronchial asthma, a common chronic respiratory disease in children, contains various inducing factors and complicated pathogenesis. Onset and development of this disease can be provoked by exposure and sensitization to allergen(s). In China, Der-f. and Der-p are the main inhaled allergens, causing pediatric allergic asthma [1].

SCIT is the recommended etiological treatment in pediatric allergic asthma, not only because of its rapid efficacy in the improvement of the symptoms but also because of its long-term effect [2, 3]. Nonetheless, the mechanism of SCIT remains unclear.

The pathogenesis of asthma has been mainly related to the imbalance of Th1/Th2 and Th17/Treg [4, 5]. Type 2 immunity is considered as the main immune type of asthma. Type 2 cytokines such as IL-4, IL-5, and IL-13 cause airway hyperresponsiveness, mucus secretion, and eosinophil aggregation and induce B lymphocyte to produce IgE-mediated allergic reaction. Th2 cells have been considered as the main source of type 2 cytokines; the imbalance of Th1/Th2 is the key to the occurrence of asthma. Besides, the immune responses of Thl7 and Treg cells also play an important role. Thl7 cells respond more strongly, and Treg expression reduced in asthma. The severity and symptom control of asthma are closely related to the imbalance of Thl7/Treg. In view of the importance of Th1/Th2 and Thl7/Treg immune imbalance in the process of asthma, it is considered that regulating Th1/Th2 and Thl7/Treg immune balance may be the mechanism of asthma treatment. Preliminary studies have shown that SCIT treatment of seasonal allergic rhinitis patients can significantly downregulate gene expression from ILC2 in the peripheral blood, and these cells can be used as serological biomarkers of desensitization efficacy [6]. These findings have opened new pathways of research for the study of specific mechanisms of action of immunotherapy.

ILC2 is an innate immunocyte involved in the occurrence and development of allergic asthma. When allergen stimulates airway epithelial cells that are impaired by asthma or the alveolar macrophages to generate IL-25 and IL-33, ILC2 is induced to proliferate and activate, through binding with corresponding receptors such as IL-25R (also called IL-17RB) and IL33R on the surfaces of ILC2, secreting type 2 cytokines such as IL-13 and IL-5, which promote airway hyperresponsiveness, eosinophilic aggregation, and mucus secretion [7]. It remains unknown whether SCIT with mite allergens would affect the regulation of ILC2 in bronchial asthma patients. This study reports observations regarding the impact of SCIT on the peripheral blood ILC2 cell expression, Th1/Th2 and Th17/Treg cell balance, and cytokine level alterations in children with mite-induced allergies.

## 2. Subjects and Methods

### 2.1. Subject Clinical Information

The study enrolled sixty outpatient kids from 5 to 10 years old with mild or moderate allergic asthma sensitized to Der-p attended from April 2017 to April 2018 in the Respiratory Department of Children's Hospital of Nanjing Medical University. All patients come from a geographic area, with similar indoor and outdoor climates. The cohort was comprised of 41 males and 19 females. All subjects were allergic to mites and had receiving antiasthmatic pharmacologic treatment for 3 months at enrollment (baseline). Children with asthma were divided into the SCIT group (*n* = 30) and the no-SCIT group (*n* = 30). The SCIT group received a combination of SCIT and pharmacologic treatment, while the no-SCIT group received only pharmacologic treatment. Asthma was diagnosed according to the GINA (Global Initiative for Asthma) updated in 2015 [8]. This study was approved by the Ethics Committee of the Children's Hospital of Nanjing Medical University, and all parents signed informed consent forms for the therapeutic regimen and experimental study arms.

The study inclusion criteria were as follows: (1) aged from 5 to 10 years old, (2) confirmed mild or moderate asthma by doctors, (3) received antiasthmatic drug treatment for three months before enrollment (baseline), (4) positive skin prick test (SPT) to Der-p was the main allergens, (5) a forced expiratory volume in 1 second (FEV1) that exceeded 70% of the predicted value, and (6) a stable physiological and psychological health status to ensure they could complete this study with minimal risk, assessed by medical history and physical examination.

The subject exclusion criteria were as follows: (1) previous treatment with specific immunotherapy, (2) FEV1 < 70%, (3) previous treatment with immunomodulators (such as spleen aminopeptide oral lyophilized powder or pidotimod oral solution) for treatment (except antiasthmatic drugs), (4) active, nonasthmatic pulmonary diseases, (5) history of anaphylactic shock or eczema of unknown origin, and (6) autoimmune diseases, mental disorders, or heart, liver, and renal diseases other than allergic reactions.

### 2.2. Therapeutic Regimen

All participants received standard pharmacologic treatments, following the recommendation of the GINA (Global Initiative for Asthma) updated in 2015 [8]. Additionally, children in the SCIT group received a standardized protocol of subcutaneous immunotherapy, Alutard SQ (Mites Allergens Alk (503) D.P). In case of the appearance of asthma attack or other allergic adverse reaction during the SCIT protocol, the treatment was discontinued or the dose was reduced.

### 2.3. Clinical Evaluation

A clinical evaluation was separately performed to the SCIT and no-SCIT groups to determine whether intergroup statistical differences between any parameters existed at baseline.

### 2.4. Total Asthma Symptom Score (TASS)

Asthma symptoms were evaluated and recorded based on the standard scoring criteria of asthma symptoms, “shortness of breath, cough, wheeze, and chest distress” [8]. The method for scoring of daytime asthma symptoms was as follows: 0 points: no symptoms; 1 point: very few symptoms, with a short duration; 2 points: two or more symptoms, with a short duration; 3 points: mild symptoms for significant time during a day, with little impact on life and work; 4 points: severe symptoms for significant time during a day, with impact on life and work; and 5 points: symptoms which were so severe that the patient could not engage in work and normal life. The method for scoring of nocturnal asthma symptoms was as follows: 0 points: no symptoms, 1 point: waking up once or early awakening, 2 points: waking up twice or early awakening, 3 points: waking up multiple times, and 4 points: difficulty of falling asleep during the evening. A summation of the total scores during daytime and nocturnal asthma symptoms was calculated as the TASS.

### 2.5. Total Medication Score (TMS)

The medication scoring system, based on the World Allergy Organization (WAO) scoring scale [9], was used to evaluate the use of antiallergic/antiasthmatic medication during 3 months preceding the study. The scoring method was as follows: 1 point: 1 tablet of oral antihistamines (loratadine 5 mg/tablet, cetirizine 5 mg/tablet), or 1 tablet of oral antileukotrienes (montelukast 5 mg/tablet) or an inhalation of *β*2 receptor agonist (salbutamol), or an inhalation of inhaled corticosteroids (budesonide, fluticasone); 2 points: low-dose inhaled steroid hormones; and 3 points: oral hormone (prednisone 5 mg/tablet).

### 2.6. Laboratory Examinations

At enrollment (baseline), 3 months and 6 months after admission, and at 8:00 a.m. on an empty stomach, all subjects had 2 mL of venous peripheral blood drawn into heparin containing tubes for anticoagulation. At each time point, the proportion of ILC2, Th1, Th2, Th17, and Treg cells in the peripheral blood and the levels of serum cytokines IL-13, IL-4, IFN-*γ*, IL-17, and TGF-*β* were measured.

### 2.7. Flow Cytometry to Detect the Percentage of Cells in the Peripheral Blood

All data collection occupied with a FACSCanto™ flow cytometer (BD Biosciences, USA), and analysis was performed with FlowJo7.6.1 (FlowJo, LLC, USA).

### 2.8. Detection of ILC2

ILC2s were detected by flow cytometry after labeling cells with lineage (anti-Human Lineage Cocktail 1; BD Bioscience, USA) and IL-17RB (R&D Systems, USA), according to the manufacturer's protocols, followed by red blood cell lysis. ILC2s were defined as lineage negative and IL-17RB positive.

### 2.9. Th1, Th2, and Th17 Detection

A human Th1/Th2/Th17 phenotyping kit (BD Biosciences, USA) was used for labeling whole blood, according to the manufacturer's protocol followed by red blood cell lysis. Th1 were defined as CD4+ IFN*γ*+ events, Th2 as CD4+IL-4+ events, and Th17 as CD4+ IL17A+ events.

### 2.10. Treg Detection

For the detection of Treg cells, whole blood cells were labeled with anti-CD4-BB515, anti-CD25-APC, and anti-CD127-PE (BD Biosciences, USA), according to the manufacturer's protocols followed by red blood cell lysis. Tregs were defined as CD4+ CD25+ CD127- events.

### 2.11. Cytometric Bead Array of Cytokines

Cytometric bead array (CBA) of cytokines was performed to detect IL-4, IL-13, and IFN-*γ* in Th1, Th2, and Th17 cells, according to the manufacturer's protocols (FACSVerse System, BD Biosciences, USA). Briefly, from 2 mL of collected whole blood, 1800 *μ*L was allocated for cytokine detection. Serum was collected from whole blood by centrifugation at 3000 rpm for 5 min. Each sample was cryopreserved at -70°C for batch analysis. For determining cytokine concentrations, standard curves were generated by serially diluting standards over 9 concentrations (1 : 1 to 1 : 256). Serum was thawed at 37°C for 30 min and centrifuged at 1500 rpm for 5 min. 50 *μ*L of serum supernatant was extracted for cytokine detection. Capture beads prepared to detect human IL-4, IL-13, and IFN-*γ* were utilized (BD Biosciences, USA).

### 2.12. ELISA for Cytokine Detection

The double antibody ELISA method was adopted for the detection of IL-17 and TGF-*β*, referring to the manufacturer's protocols (Westang Biotechnology Ltd., Shanghai, China).

### 2.13. Statistical Analysis

Statistical significance was assessed with Student's *t*-test. A *P* value of <0.05 was considered significant.

## 3. Results

Each treatment group (no-SCIT group or SCIT group) contained 30 subjects. There was no intergroup statistical difference regarding sex, age, asthma symptom score, or drug scores at baseline (Table 1). No one took stimulants during observation. There was no loss of follow-up during the study.

### 3.1. Comparison of TASS and TMS

**Figure 1:**
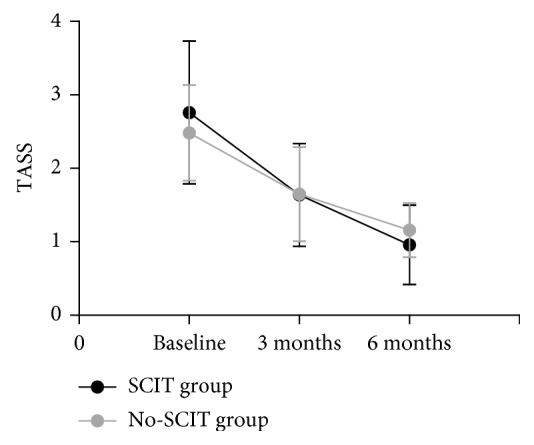
Changes in TASS in the SCIT and no-SCIT groups were compared over the three time points.

There was no significant difference in TASS between groups at any time point. However, both groups induced a significant and progressive reduction in TASS after 3 and 6 months of treatment compared with baseline (*P* < 0.05) (Figure 1).

**Figure 2:**
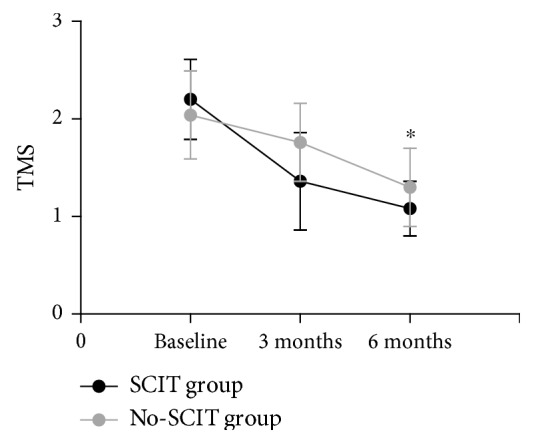
Changes in TMS in the SCIT and no-SCIT groups were compared over the three time points. ^∗^Designates a *P* < 0.05 intergroup difference.

TMS in the SCIT group decreased significantly after 3 months and 6 months of treatment compared with baseline with statistical significance (*P* < 0.05). There was no statistical difference in TMS in the no-SCIT group at 3 months of treatment, but there was a significant difference after 6 months of treatment compared with baseline (*P* < 0.05). After 3 months and 6 months of treatment, the change of clinical medication in the SCIT group was more obvious than that in the no-SCIT group, and there was a statistical difference (*P* < 0.05) (Figure 2).

### 3.2. Comparison of ILC2 Percentage and IL-13 Level

**Figure 3:**
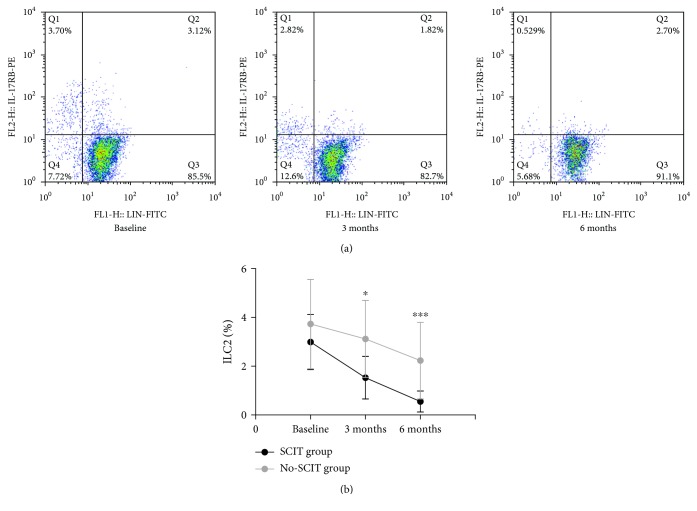
Changes in ILC2 percentage. (a) Flow cytometry was used to detect the percentage of ILC2 cells by labeling peripheral blood with antilineage and IL-17RB (LIN^−^ IL-17RB^+^) fluorescent antibodies of subjects in the SCIT group at three time points; (b) changes in ILC2 percentage were calculated from flow cytometry data for subjects in the SCIT and no-SCIT groups and plotted for the three time points. ^∗^Designates a *P* < 0.05 intergroup difference; ^∗∗∗^designates a *P* < 0.001 intergroup difference.

**Figure 4:**
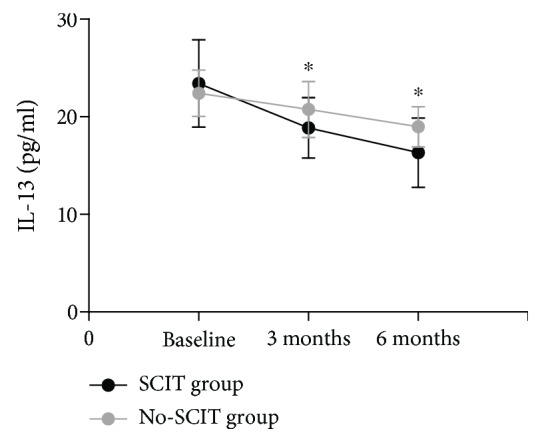
Intergroup comparison of IL-13 concentration for children in the SCIT and no-SCIT groups. ^∗^Designates a *P* < 0.05 intergroup difference.

There was no significant difference in ILC2 percentage or IL-13 level between groups at baseline. However, SCIT induced a significant and progressive reduction in ILC2 percentage and IL-13 level after 3 and 6 months of treatment compared with baseline that was significantly higher than the no-SCIT treatment group (*P* < 0.05) (Figures 3 and 4).

### 3.3. Comparison of Th1/Th2 and IFN-*γ*/IL-4 Ratios

**Figure 5:**
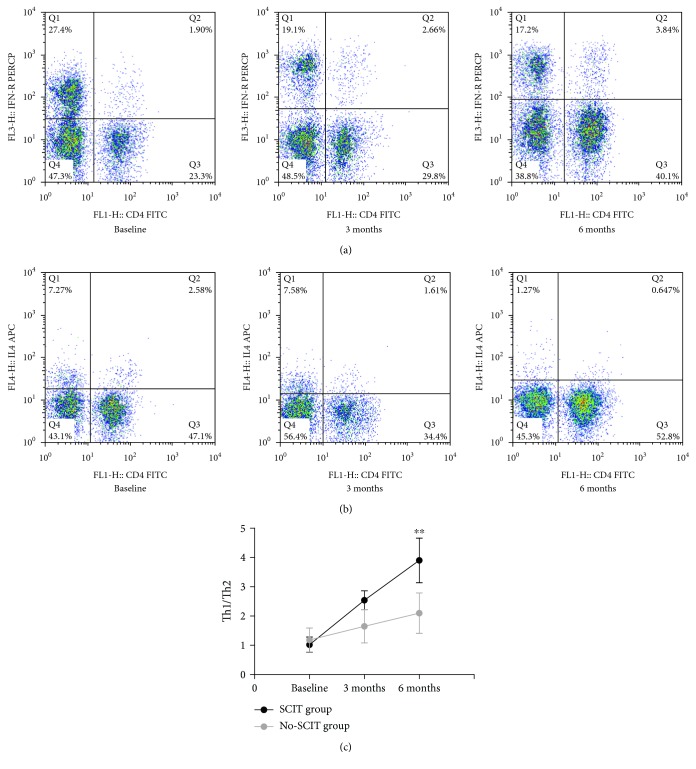
Changes in peripheral blood Th1/Th2 cell ratios. Flow cytometry was used to detect (a) the percentage of Th1 cells in peripheral blood by labeling with anti-CD4 and IFN-*γ* (CD4^+^IFN-*γ*^+^) fluorescent antibodies, (b) the percentage of Th2 cells using anti-CD4 and IL-4 (CD4^+^IL-4^+^) fluorescent antibodies for subjects in the SCIT group at three time points; (c) changes in the Th1/Th2 ratio for the SCIT and no-SCIT groups at the three time points. ^∗^Designates *P* < 0.05 intergroup difference; ^∗∗^designates a *P* < 0.01 intergroup difference.

The Th1/Th2 ratio increased gradually during treatment for both groups, and SCIT induced a significant and progressive growth in the Th1/Th2 ratio after 6 months of treatment compared with baseline that was significantly higher than the no-SCIT treatment group (*P* < 0.01) (Figure 5).

**Figure 6:**
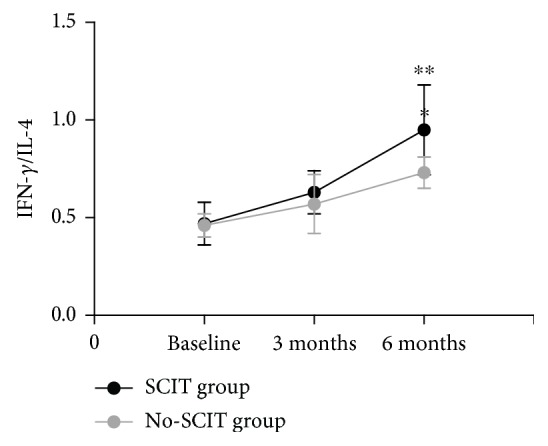
Changes in the IFN-*γ*/IL-4 ratio in the SCIT and no-SCIT groups were compared over the three time points. ^∗∗^designates a *P* < 0.01 intergroup difference.

The IFN-*γ*/IL-4 ratio increased gradually during treatment for both groups, and SCIT induced a significant and progressive growth in the IFN-*γ*/IL-4 ratio after 6 months of treatment compared with baseline that was significantly higher than the no-SCIT treatment group (*P* < 0.01) (Figure 6).

### 3.4. Comparison of the Th17/Treg and IL-17/TGF-*β* Ratios

**Figure 7:**
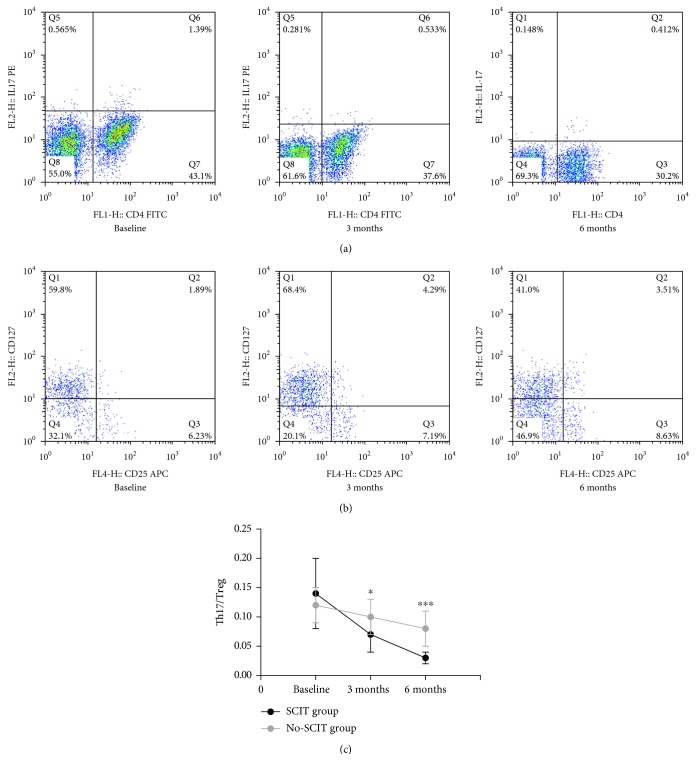
Changes in Th17 and Treg cell percentage and Th17/Treg ratio of children in both groups before and after treatment. (a) Flow cytometry was used to detect the percentage of Th17 cells by labeling peripheral blood with anti-CD4 and IL-17 (CD4^+^ IL-17^+^) fluorescent antibodies of subjects in the SCIT group at three time points; (b) Treg cells were similarly detected with anti-CD4, CD25, and CD127 (CD4^+^ CD25^+^ CD127^−^) antibodies; (c) changes in the Th17/Treg ratio were calculated from flow cytometry data for subjects in the SCIT and no-SCIT groups and plotted for the three time points. ^∗^Designates a *P* < 0.05 intergroup difference; ^∗∗∗^designates a *P* < 0.001 intergroup difference.

The Th17/Treg ratio decreased gradually during treatment for both groups, and SCIT induced a significant and progressive reduction in the Th17/Treg ratio after 3 months and 6 months of treatment compared with baseline that was significantly higher than the no-SCIT treatment group (*P* < 0.05)(Figure 7).

**Figure 8:**
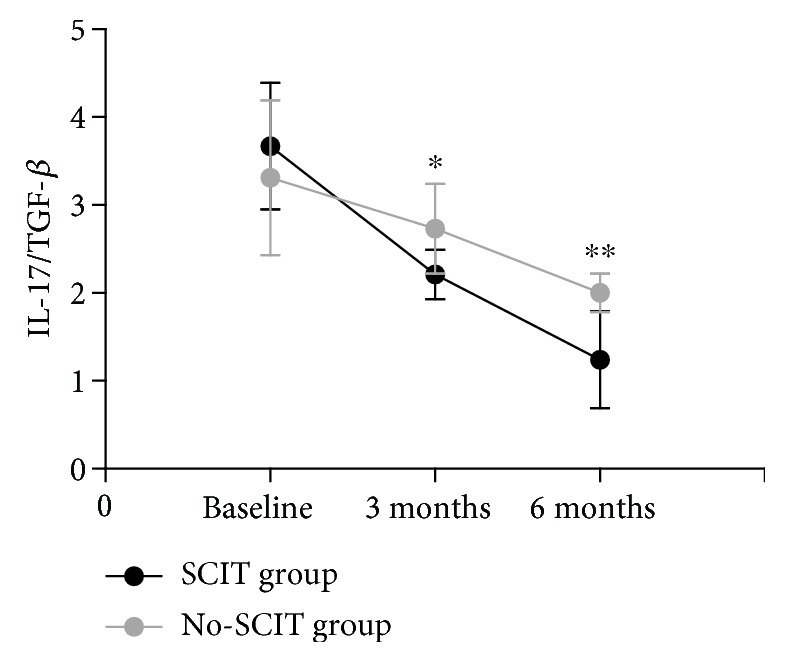
Changes in the IL-17/TGF-*γ* ratio in the SCIT and no-SCIT groups were compared over the three time points. ^∗^designates a *P* < 0.05 intergroup difference; ^∗∗^designates a *P* < 0.01 intergroup difference.

The IL-17/TGF-*β* ratio decreased gradually during treatment for both groups, and SCIT induced a significant and progressive reduction in the Th17/Treg ratio after 3 months and 6 months of treatment compared with baseline that was significantly higher than the no-SCIT treatment group (*P* < 0.05) (Figure 8).

## 4. Discussion

Increased exposure to pollution and allergens in the current lifestyle has been linked to an increase in the incidence of asthma in children and adults. Prevention of allergen exposure, early detection of allergen sensitization, or prescription of targeted allergen immunotherapy are effective methods of reducing asthma development. Pharmacologic treatment improves asthma symptoms safely but has not changed the natural evolution of the disease. Comprehensive treatment including specific immunotherapy should be adopted for asthma.

Importantly, numerous studies have confirmed the long-term efficacy of SCIT [10]; it may reduce the risk of asthma for several years after treatment discontinuation [11]. In addition, SCIT can change the course of the disease by preventing the emergence of new sensitizations and has a rapid onset similar to standard drug treatment [12].

Type 2 innate lymphoid cells (ILC2s) are a new member of the innate lymphoid cell family which has been recently discovered. The type 2 cytokines, interleukin- (IL-) 4, IL-5, IL-9, and IL-13, are the major drivers of allergic asthma and studies. Type 2 innate lymphoid cells (ILC2s) provide a potent early innate source of the cytokines IL-5 and IL-13. Even though it is categorized as an innate immune cell, ILC2 can mediate acquired immunity. By producing type 2 cytokines [13], ILC2 proves to be a fresh option to induce airway hyperreactivity (AHR), mucus secretion, and eosinophilic aggregation and promote B lymphocytes to generate IgE, which is possible to exacerbate asthma symptoms. Research confirmed that SCIT to patients with seasonal allergic rhinitis can reduce ILC2 percentage in the peripheral blood, comparing to patients who did not receive subcutaneous desensitization [6].

The current study is aimed at identifying differences in ILC2 and associated cytokines between SCIT and no-SCIT groups with asthma over time. The data confirmed that in addition to the effects of improving asthma symptoms, SCIT reduced the proportion of ILC2 in the peripheral blood of children with asthma and downregulated the level of IL-13, a cytokine secreted from ILC2 cells among others. Furthermore, the results showed that the percentage of the ILC2 cells in the peripheral blood progressively decreased during the SCIT treatment with greater magnitude than that in the no-SCIT group. By sustaining a diminishing of the effect of ILC2, SCIT improves strong ability to asthma.

Previous in vitro studies have revealed that ILC2 plays an important role in the tight junction of epithelial cells of bronchi and airways as well as barrier functions, which depend on the production of the cytokine IL-13 [14]. Indeed, the expression level of IL-13 producing ILC2 (IL-13+ ILC2) is negatively correlated with the degree of asthma control. As asthma control progresses, the expression of IL-13 by ILC2 cells moves gradually closer to normal levels [15]. The data in this study supports the hypothesis that SCIT improves allergic symptoms by mediating ILC2, which may alter IL-13 production. However, IL-13 is also generated by Th2 cells, and over the treatment period of this study, the percentage of Th2 cells also decreased. Therefore, it is impossible to discern the extent to which ILC2 or Th2 alterations contributed to IL-13 decreases.

A disequilibrium or imbalance of Th1/Th2 cells remains a common hypothesis of asthma pathogenesis and desensitization treatment mechanisms. This study found that SCIT increased the Th1/Th2 cell and the IFN-*γ*/IL-4 cytokine ratios. Secreted specifically by Th1 cells, IFN-*γ* promotes the activation of Th1 cells, inhibits the activation of Th2, lowers the synthesis and secretion of IgE, reduces inflammation, and relieves the symptoms of many asthma patients. On the other hand, IL-4 from Th2 cells promotes proliferation of Th2 cells and expression of intravascular endothelial cell adhesion molecules that induce monocyte, eosinophil, and lymphocyte aggregation at inflammatory sites, improves the effects of B cells and T cells, promotes the humoral immune response, and aggravates allergic reactions. The change of the IFN-*γ*/IL-4 ratio reported in this study supports the premise that SCIT promotes a Th1/Th2 immunologic balance, consistent with the early clinical studies [16, 17].

The data also showed that SCIT immunoregulation of Th1 and Th2 cells was mainly realized through the downregulation of Th2, since intergroup comparison did not show differences in the percentage of peripheral blood Th1 throughout the treatment. Further, the intergroup difference in Th1 IFN-*γ* was static while the intergroup difference in Th2 IL-4 decreased over time, indicating that SCIT downregulated the level of IL-4 mainly through the inhibition of the Th2 cell expression.

Th17 and Treg cells maintain a complicated relationship regarding specific immunotherapy. The cells are functional antagonists but show coordinated differentiation. Normally, the immunologic balance of Th17 and Treg cells helps to maintain homeostasis of the body [18]. Th17 produces IL-17, and in asthma, this inflammatory reaction may be upregulated [19]. Treg cells effectively inhibit this immunoreaction to maintain the body's immune system equilibrium. Also, they mediate immune tolerance of autoantigen and block the generation of pathological inflammatory reactions and autoimmune response. The functions of Th17 are inhibited in children with asthma [20]. Tregs secrete TGF-*β*, which inhibits the activation of inflammatory cells through negative feedback. Th17/Treg disequilibrium in asthma patients is closely associated with the severity of asthma [21]. This study has shown that during the SCIT treatment, the Th17 cell percentage decreased, Treg cell percentage increased, and the Th17/Treg and IL-17/TGF-*β* ratio decreased. These data further validate the hypothesis that the subcutaneous immunotherapy could regulate the Th17/Treg equilibrium.

This study does have some limitations. First, this was an unblinded prospective study without randomization. Second, we did not assess the clinical efficacy of SCIT on asthma symptoms and the use of drug; therefore, it cannot conclude to what extent changes in ILC2 or T helper cell subsets were associated with symptoms. Thus, further studies are needed to determine whether ILC2 and T helper cell populations can serve as a biomarker for efficacy of SCIT in allergic asthma. Third, cosensitization is common in allergic individuals, but we did not assess the level of cosensitization in our population. Fourth, we also did not assess allergen-specific Th1/Th2 population levels, which could exhibit changes different from global ones.

Taken together, the data support and enhance previous hypotheses explaining the mechanism of SCIT suppression of asthma. First, in this study, SCIT seems to inhibit the expression of peripheral blood ILC2. Second, imbalances in T lymphocyte subgroups manifest as increased Th1/Th2 and decreased Th17/Treg ratios, which SCIT appears to resolve. Generally, the data shows SCIT improvement to the magnitude and rate of blood cellular and cytokine changes that mitigate asthma effects in children when compared to standard drug treatment. To better understand the changes in cell ratios and cytokine levels, further tests are essential to identify the relevant cellular pathways and interactions involved.

## Figures and Tables

**Table 1:** Subject demographics (*x* ± *s*, *n* = 30).

	SCIT group	No-SCIT group	*t*	*P* value
Gender (male/female)	21/9	20/10	0.077	0.781
Age (years)	6.25 ± 1.01	6.12 ± 1.10	0.274	0.784
Asthma symptom score	2.76 ± 0.97	2.48 ± 0.65	0.889	0.374
Medication score	2.20 ± 0.41	2.04 ± 0.45	1.255	0.209

Note: data were expressed as mean ± standard deviation.

## Data Availability

No data were used to support this study.
